# Shoaling reduces metabolic rate in a gregarious coral reef fish species

**DOI:** 10.1242/jeb.139493

**Published:** 2016-09-15

**Authors:** Lauren E. Nadler, Shaun S. Killen, Eva C. McClure, Philip L. Munday, Mark I. McCormick

**Affiliations:** 1 College of Marine and Environmental Sciences, James Cook University, Townsville, Queensland 4811, Australia; 2 ARC Centre of Excellence for Coral Reef Studies, James Cook University, Townsville, Queensland 4811, Australia; 3 Institute of Biodiversity, Animal Health and Comparative Medicine, University of Glasgow, Glasgow G12 8QQ, UK

**Keywords:** Calming effect, Metabolism, Body condition, Respirometry, Energetics, *Chromis viridis*

## Abstract

Many animals live in groups because of the potential benefits associated with defense and foraging. Group living may also induce a ‘calming effect’ on individuals, reducing overall metabolic demand. This effect could occur by minimising the need for individual vigilance and reducing stress through social buffering. However, this effect has proved difficult to quantify. We examined the effect of shoaling on metabolism and body condition in the gregarious damselfish *Chromis viridis*. Using a novel respirometry methodology for social species, we found that the presence of shoal-mate visual and olfactory cues led to a reduction in the minimum metabolic rate of individuals. Fish held in isolation for 1 week also exhibited a reduction in body condition when compared with those held in shoals. These results indicate that social isolation as a result of environmental disturbance could have physiological consequences for gregarious species.

## Abstract (precis)

**Highlighted Article:** Group living reduces metabolic rate in a shoaling damselfish species, while isolation reduces body condition. Social isolation due to environmental disturbance may therefore have physiological consequences for gregarious species.

## INTRODUCTION

Group living is widespread among animal species and carries a number of benefits (Krause and Ruxton, 2002). It has been shown, for example, that individuals within groups can reduce their energy expenditure in a variety of situations, including the costs of flight in birds (Weimerskirch et al., 2001), swimming in fish (Hemelrijk et al., 2015), web-building in spiders (Jakob, 1991) and thermoregulation in mice (Scantlebury et al., 2006). Individuals may also be able to reduce overall metabolic demand through group living as a result of a ‘calming effect’ (Martin et al., 1980; Parker, 1973; Trune and Slobodchikoff, 1976). One factor that likely contributes to this effect is a reduced need for individual vigilance, as animal groups exhibit improved threat detection by having ‘many eyes’ to scan for predators (Roberts, 1996; Ward et al., 2011). Individuals accustomed to a social environment may also exhibit reduced stress when allowed to associate with conspecifics (Hennessy et al., 2009).

A number of methods have thus far been employed to estimate the magnitude of the calming effect in a range of gregarious fish species. First, the ventilation rate of shoaling versus solitary individuals has been recorded to estimate metabolic rate (Lefrancois et al., 2009; Queiroz and Magurran, 2005). A second method uses respirometry (where oxygen uptake is measured as a proxy for aerobic metabolism) to compare the sum of each individual's metabolic rate when measured alone with the metabolic rate of the shoal measured together (Parker, 1973; Schleuter et al., 2007). Although these methods have provided supporting evidence for the calming effect, they do not directly measure social influences on individual physiology. Lastly, a third method has been employed, in which cues of conspecifics are presented to a solitary individual either by allowing conspecifics to freely move around the respirometry chamber (Plath et al., 2013) or by placing individuals in neighbouring respirometry chambers (Herskin, 1999). However, this method has not detected evidence of a calming effect, suggesting that this methodology may fail to sufficiently simulate shoaling conditions to elicit one. A calming effect may not be detected if the shoal-mates move too far away from the focal individual or if olfactory cues of shoal-mates are too weak to allow social recognition (Brown and Smith, 1994; Herskin, 1999; Plath et al., 2013; Ward et al., 2002).

In this study, we developed a novel method to measure the calming effect's influence on body condition and metabolic rate in a gregarious coral reef fish. Fish were held for 2 weeks either alone or in a shoal before measurement of metabolic rate. Metabolic rate of solitary versus shoaling individuals was then tested using custom respirometry chambers that were designed to provide visual and olfactory cues of shoal-mates to a focal individual. We hypothesised that individuals housed in shoals and tested with shoal-mates would exhibit the greatest body condition, lowest minimum metabolic rate and reduced physiological reaction to stress compared with individuals in solitary treatments.

## MATERIALS AND METHODS

### Fish collection and maintenance

This experiment was conducted at the Lizard Island Research Station (LIRS; a facility of the Australian Museum) in the northern Great Barrier Reef (14°40′08″S, 145°27′34″E), using a gregarious damselfish species, the blue-green puller, *Chromis viridis* Cuvier 1830. Shoals of juvenile *C. viridis* (standard length=3.69±0.03 cm, wet mass=1.84±0.04 g; means±s.e.m.) were collected from reefs adjacent to LIRS using hand nets and barrier nets. Fish were either placed into shoals of 10 individuals (shoal holding treatment, *n*=8) or held in isolation (solitary holding treatment, *n*=8) at a stocking density of 1 fish l^−1^. Fish were fed a body-mass-specific diet twice daily with INVE Aquaculture pellets and newly hatched *Artemia* spp.

### Ethics

This research was conducted under James Cook University Animal Ethics approval number A2103.

### Body condition measurement

Focal individuals were chosen at random and tagged with visible implant elastomer (Northwest Marine Technology, Tumwater, WA, USA) so they were identifiable over time (Hoey and McCormick, 2006). Each holding treatment (solitary, shoal) was maintained for 2 weeks under a natural light cycle (14 h:10 h light:dark). At three time points during this period (weeks 0, 1 and 2), focal fish were measured for wet mass (*M*; ±0.0001 g) and standard length (*L*; ±0.01 cm), from which Fulton's *K* condition factor [*K*=100×(*M/L*^3^)] was calculated.

### Respirometry

**Fig. 1.**
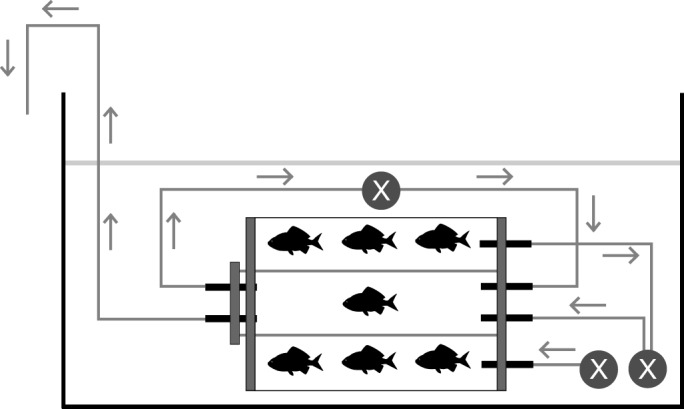
**Side view of the respirometry chamber.** The experimental setup was composed of an inner respirometry chamber (length=13.5 cm, inner diameter=3.24 cm, volume of chamber and associated gas-impermeable tubing=100 ml) and an outer shoal-mate holding chamber (length=12.0 cm, inner diameter=11.4 cm, volume=1.10 litres). Arrows indicate the direction of water flow through tubing. Grey circles marked with an X indicate water pumps used for mixing the inner chamber and flushing both chambers.

Metabolic rate was measured for focal fish using custom respirometry chambers composed of two cylindrical glass tubes (inner respirometry chamber and outer shoal-mate holding chamber) with acrylic end caps and immersed in separate, temperature-controlled water baths (29±0.5°C; Fig. 1). All individuals from both treatments were retrieved from holding tanks using plastic tubs to minimise capture time and eliminate air exposure. Focal fish were then placed in 1 litre plastic bags filled with seawater for ∼10 min prior to transfer to the inner respirometry chamber, in order to allow focal fish to recover from the capture protocol. The inner respirometry chamber was connected to a recirculating pump, to mix water in the respirometer, and a flushing pump, to flush the chamber with oxygen-saturated water for 3 min between each 9 min measurement period. The timing of this flushing and measurement cycle ensured that oxygen saturation in the inner chamber remained above 80% air saturation at all times (Hughes, 1973). The outer chamber was affixed to the exterior of the inner chamber, to provide visual and olfactory cues of shoal-mates to the focal individual; this chamber was aerated with a continuously running flush pump and the water leaving the outflow port was attached to the in-flow vent for the inner chamber's flush pump, in order to provide the shoal-mates' olfactory cues to the focal individual. Water mixing from the two chambers was confirmed with preliminary tests using food colouring. To ensure that the inner chamber was being flushed with equally oxygenated water in both testing treatments, the flush pump utilised a mixture of the outflow water from the outer chamber and ambient water from the surrounding aquarium. The diameter of this outer chamber prevented shoal-mates from swimming >1.5 body lengths from the focal individual.

Dissolved oxygen concentration in the inner chamber was measured every 2 s using a Fire-Sting fibre-optic oxygen meter (Pyroscience, Germany) connected to a computer. The oxygen-sensing optode was mounted in the recirculation loop in a flow-through cell (Svendsen et al., 2016). Focal fish were starved for 24–25 h prior to experimentation to ensure that they were in a post-absorptive state (Niimi and Beamish, 1974) and were left undisturbed in the respirometers for 11–12 h overnight, as *C. viridis* is quiescent at night. Preliminary studies of *C. viridis* run for 36 h indicated that oxygen consumption stabilised within ∼5 h, with the lowest levels achieved overnight (S.S.K., L.E.N. and M.I.M., unpublished data). This was consistent with the data presented here (average time to stabilise=4.6 h, and was not significantly different between treatments, *P*>0.05). A dim light remained on through the night in the laboratory, allowing the focal fish to see their shoal-mates in shoal testing trials. Slopes (*s*) were calculated from plots of oxygen concentration versus time using linear least squares regression (LabChart v6 software) and converted to rate of oxygen uptake (*Ṁ*_O_2__; mg O_2_ h^−1^), excluding the first and last minute of each measurement period to allow the oxygen concentration in the recirculation loop to stabilise following the flushing period. All *r*^2^ values were greater than 0.97. Bacterial respiration was measured in empty chambers for three measurement periods before and after trials and was then subtracted from all fish respiration measurements, assuming a linear increase in bacterial respiration over time (Rodgers et al., 2016).

The metabolic rate of each focal fish was recorded in a solitary (no shoal-mates in the outer chamber) and a shoal testing treatment (six shoal-mates in the outer chamber). Three measures of metabolic rate were analysed. First, minimum measured metabolic rate in fish exposed to each treatment (MR_min_) was estimated using the protocol typically employed to measure standard metabolic rate (the metabolic rate of a resting ectotherm) in the literature. This was accomplished by taking MR_min_ as the lowest 10th percentile of *Ṁ*_O_2__ measurements (Chabot et al., 2016; Killen, 2014) and comparisons were drawn between individuals tested alone and with a shoal. Second, routine metabolic rate (RMR, the metabolic rate of an undisturbed animal including costs of random activity) was calculated as the mean *Ṁ*_O_2__ excluding the first 5 h in the respirometer. Differences between fish tested alone (RMR_alone_) and fish tested in shoals (RMR_shoal_) were assessed (Killen et al., 2011). Third, the initial stress response (ISR) was taken as the difference between the initial metabolic rate (first slope following transfer to the respirometer) and MR_min_. *Ṁ*_O_2__ is commonly used as an indicator of stress and reaction to threats such as predation, because of the previously established link between oxygen uptake and stress hormones, including cortisol and epinephrine (e.g. Brown et al., 1982; Morgan and Iwama, 1996). In this study, the stressor was the handling stress induced during transfer to the respirometer and any stress of being in isolation.

### Statistical analyses

All statistical analyses were conducted in R v.3.2.4, using package ‘nlme’ (http://CRAN.R-project.org/package=nlme). Differences in body condition over time were assessed using a general linear mixed-effects model (GLMM) corrected for autocorrelation, with holding treatment (shoal or solitary) and time (weeks 0, 1, 2) as fixed effects and individual as a random effect. Differences in the MR_min_, RMR and ISR were analysed using a GLMM with holding pattern (shoal or solitary) and testing pattern (shoal or solitary) as fixed effects, body mass as a covariate (to account for differences in body size) and individual as a random effect.

## RESULTS AND DISCUSSION

**Fig. 2.**
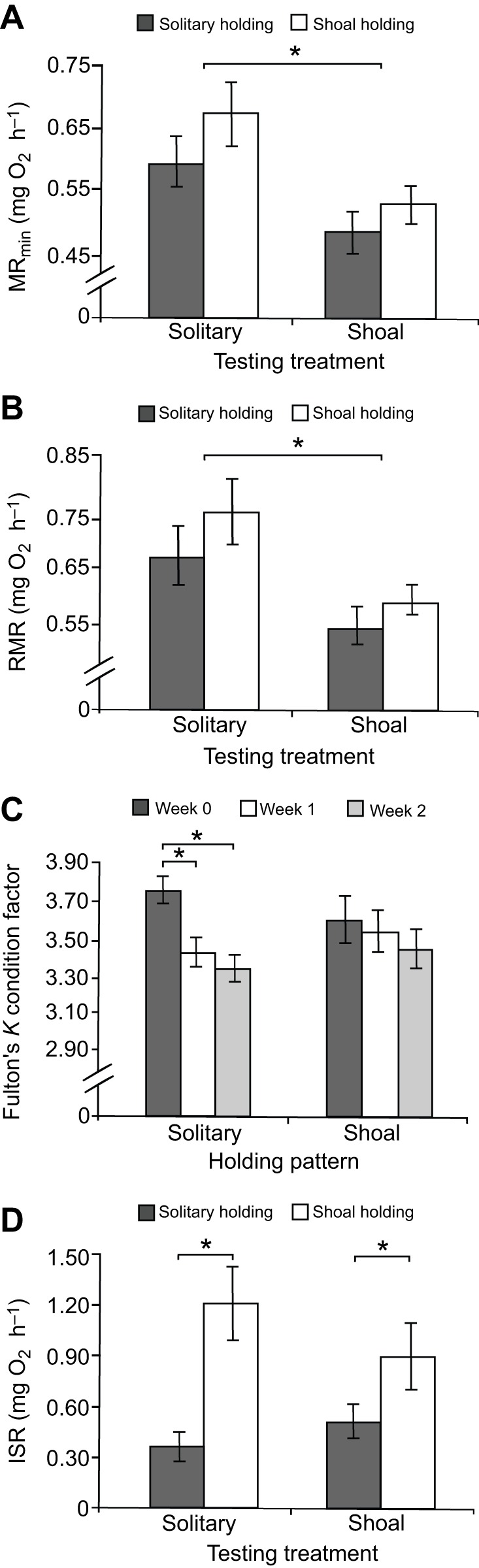
**Effects of shoaling on the body condition and metabolism of *Chromis viridis*.** Effect of holding and testing treatment on (A) minimum metabolic rate (MR_min_, mg O_2_ h^−1^) and (B) routine metabolic rate (RMR; mg O_2_ h^−1^). (C) Effect of holding treatment on individual body condition (Fulton's *K* condition factor). (D) Effect of holding and testing treatment on the initial stress response (ISR; mg O_2_ h^−1^). Metabolic rate measures were mass-corrected using residuals of the relationship between log body mass and log metabolic rate added to the fitted value for mass=1.84 g, the mean mass of all fish used in the study. Error bars are s.e.m. and *n*=8 for all treatments. Asterisks indicate statistical significance (*P<0.05).

The results suggest that the minimal estimated metabolic rate of gregarious species may be higher when individuals are measured alone versus when they are measured in a shoal, potentially because of an increase in energy spent on vigilance or an autonomic stress response to social isolation (Barreto and Volpato, 2011; Hennessy et al., 2009; Roberts, 1996). MR_min_ of fish tested in a shoal was significantly lower than MR_min_ of solitary fish, with an average reduction of 25.9% (5–60% range; GLMM: *F*_1,14_=27.27, *P*=0.0004; Fig. 2A). Similar results were also found for RMR, with RMR of fish in a shoal significantly lower than RMR of solitary fish (GLMM: *F*_1,14_=17.34, *P*=0.0019; Fig. 2B). Respirometry treatment had a comparable effect on individuals from both the solitary and shoal holding treatments (MR_min_ GLMM: *F*_1,14_=1.26, *P*=0.2812; RMR GLMM: *F*_1,14_=1.14, *P*=0.3033).

Holding treatment did have a significant impact on body condition. Individuals that were kept alone in their holding tanks exhibited a reduction in condition factor from week 0 to 1 (GLMM: *F*_1,30_=9.16, *P*=0.0050; Fig. 2C). The measured increase in MR_min_ in the solitary testing treatment likely contributed to this reduction in body condition. As feeding rate can decrease immediately following social isolation in gregarious species (Barreto and Volpato, 2011), reduced food intake in the solitary holding treatment may have compounded this effect.

Individuals accustomed to the shoal holding treatment exhibited a stronger physiological reaction to handling stress during transfer to the respirometer than those acclimated to an isolated condition. ISR was more than double in focal individuals that had been held in shoals as compared with individuals held alone (*F*_1,14_=9.62, *P*=0.0078; Fig. 2C), regardless of whether fish were measured for MR_min_ in a shoal or in isolation (*F*_1,14_=0.21, *P*=0.6559; Fig. 2D). Plath et al. (2013) found a similar trend in shoaling minnows, in which oxygen consumption rate increased immediately following exposure to a shoal testing treatment. Individuals that were held in shoals but measured for metabolic rate in isolation exhibited elevated ISR likely because of the stress of acute social isolation, which can increase circulating glucocorticoids in gregarious species (Galhardo and Oliveira, 2014; Lyons et al., 1993). Fish in the solitary holding treatment may have grown accustomed to being alone, relying less on the presence of shoal-mates for risk assessment and stress reduction. As fish held alone would not have had shoal-mates to aid in vigilance, they may have increased the threshold of threat at which they instigate a stress response, which could explain their lower ISR. However, further studies quantifying the role of individual vigilance in the calming effect would be essential to tease apart this mechanism (Roberts, 1996).

Many factors may influence the magnitude of the calming effect in social species, such as the degree of social organisation, ontogenetic stage and novelty of the environment (Hennessy et al., 2009). Therefore, further studies should investigate whether the calming effect is maintained under different conditions. In highly territorial species, the presence of conspecifics can increase metabolic demand and aggressive behaviours (Killen et al., 2014; Sloman et al., 2000), highlighting the importance of behavioural traits in physiological responses to conspecifics. In addition, many of the benefits of group living increase up to an optimal shoal size, including vigilance and foraging (Fischer et al., 2015; Goldenberg et al., 2014; Magurran and Pitcher, 1983). Therefore, the magnitude of the calming effect is likely to vary depending on the group size presented during testing. Lastly, as recent studies indicate evidence of intraspecific variation in sociability (Hennessy et al., 2009; Killen et al., 2016), the adaptive value of group living to fishes may vary among individuals because of differences in physiological and behavioural characteristics.

In an ecological context, disturbances such as storms and flooding can lead to group disruption and forced social isolation in animal communities (e.g. Lassig, 1983; Yoon et al., 2011). The results of this study suggest that solitary members of gregarious species may experience increased physiological reactions to stress and energy expenditure. An autonomic stress response owing to social isolation could have a range of additional repercussions for social species, with implications for overall fitness (Hennessy et al., 2009).
